# Bilateral Single-Stage Nephrectomy for Synchronous Bilateral Renal Cell Carcinoma

**DOI:** 10.15586/jkcvhl.v8i1.151

**Published:** 2021-01-25

**Authors:** Ahmed Kotb, Amer Alaref, David Kisselgoff, Asmaa Ismail, Radu Rozenberg, Nishigandha Burute, Walid Shahrour, Owen Prowse, Hazem Elmanasy

**Affiliations:** 1 Urology Department, Thunder Bay Regional Health Science Centre, Northern Ontario School of Medicine, Thunder Bay, ON, Canada;; 2 Radiology Department, Thunder Bay Regional Health Science Centre, Northern Ontario School of Medicine, Thunder Bay, ON, Canada

**Keywords:** RCC, Lumbotomy, nephrectomy

## Abstract

Bilateral synchronous renal cell carcinoma (RCC) is uncommonly encountered. Debate exists among urologists in managing these cases in a single surgery versus staged surgeries. We aim to report our experience in managing encountered cases using single-stage surgeries. Retrospective collection of cases with pathologically confirmed RCC that had single-stage bilateral renal surgery over the past 2 years. Three cases were identified. Patients were managed using bilateral transverse lateral lumbotomy. All patients did not have intraoperative or postoperative complications. Kidney function stayed stable after surgery. Single-stage bilateral renal surgery is a safe procedure. Bilateral transverse lateral lumbotomy allows for a fast and safe surgery with minimal complications. There is a possible histological dis-concordance in bilateral synchronous RCC.

## Introduction

The incidence of most cancers is on the rise. Canadian cancer statistics was expecting 7500 new kidney cancer diagnosis within 2020 with an age standardized incidence rate of 17.3 in 100,000 population, with Ontario expected to have one-third of the newly diagnosed cases (1). Bilateral renal cell carcinoma (RCC) occurs in less than 5% of kidney cancer, with the incidence of synchronous bilateral RCC being 1 out of 333 (0.3%) patients with RCC (2).

The aim of our work was to present our experience in managing synchronous bilateral RCC using single-stage bilateral transverse lateral lumbotomy incision.

## Methods

Retrospective data analysis of patients having synchronous bilateral clinically suspected RCC, which was managed by single-stage surgery by a single urologist (AK), was the method of choice. The urologist performing the surgery had 15 years’ experience in performing renal cancer surgery. Data collection included information on patients’ age, sex and clinical presentation, preoperative eGFR and images, pathological analysis, and postoperative eGFR. All cases included in the study were managed by open surgery. All had single-stage bilateral transverse lateral lumbotomy incision, an approach we published last year (3). During surgery, the patient is positioned with the side of surgery elevated to 45 degree. An upward smiling incision subcostal and running parallel to the costal margin is done. Direct adequate access to the kidney could be done without the need for extensive diaphragmatic dissection. Full mobilization of the kidney and renal pedicle is done. The tumor is then identified and marked all around, few millimeters away from the tumor edge with cautery. We then clamp the pedicle and complete the surgery under warm ischemia. All patients had their first postoperative computed tomography scan 12 months post-surgery.

Our study included only patients that had single-stage bilateral renal surgery. Institutional ethical approval as well as patients’ consent to be included in the study was obtained.

## Results

We could identify three patients managed by single-stage bilateral renal surgery for clinically suspected RCC. All three patients were men with a median age of 76 years. They had their cancer diagnosed accidentally without symptoms suggestive of renal tumor.

**Figure 1:**
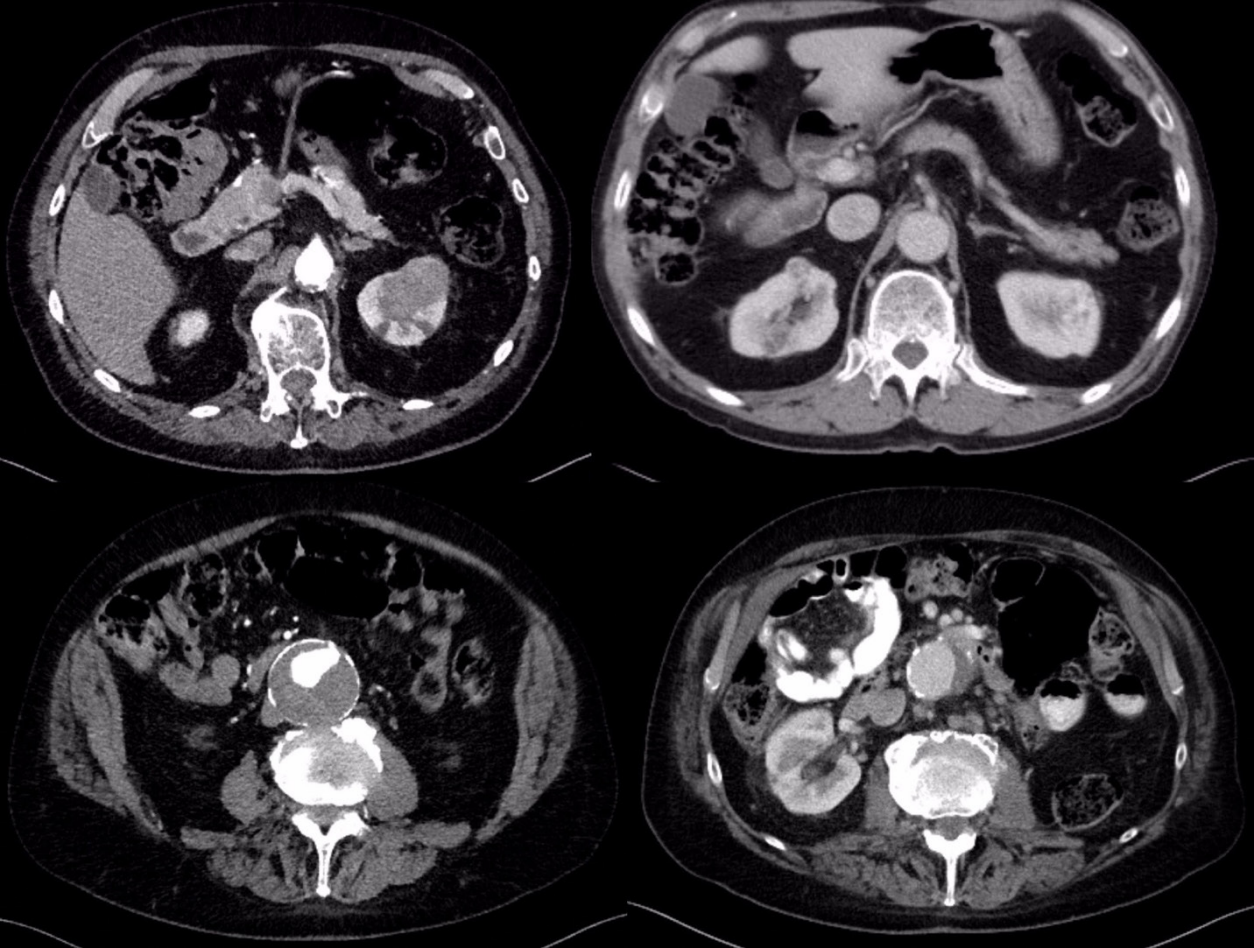
First patient: preoperative bilateral renal tumor, large AAA, and 14 months’ postoperative image showing no recurrence.

The first patient was 78 years old and was being followed by the vascular surgery team for a large abdominal aortic aneurysm (AAA), with bilateral enhancing renal masses diagnosed at the time of angiography. The right kidney had a single mass while the left kidney had multifocal tumors. The patient had single-stage left radical nephrectomy (RN) and right partial nephrectomy (PN). Pathology showed right clear-cell RCC (ccRCC) and left papillary RCC (pRCC) with negative margins. His eGFR was stable at 14 months after surgery and a new scan 1-year post surgery confirmed no cancer recurrence. Figure 1 shows the bilateral renal tumor, AAA, and a postoperative scan.

**Figure 2:**
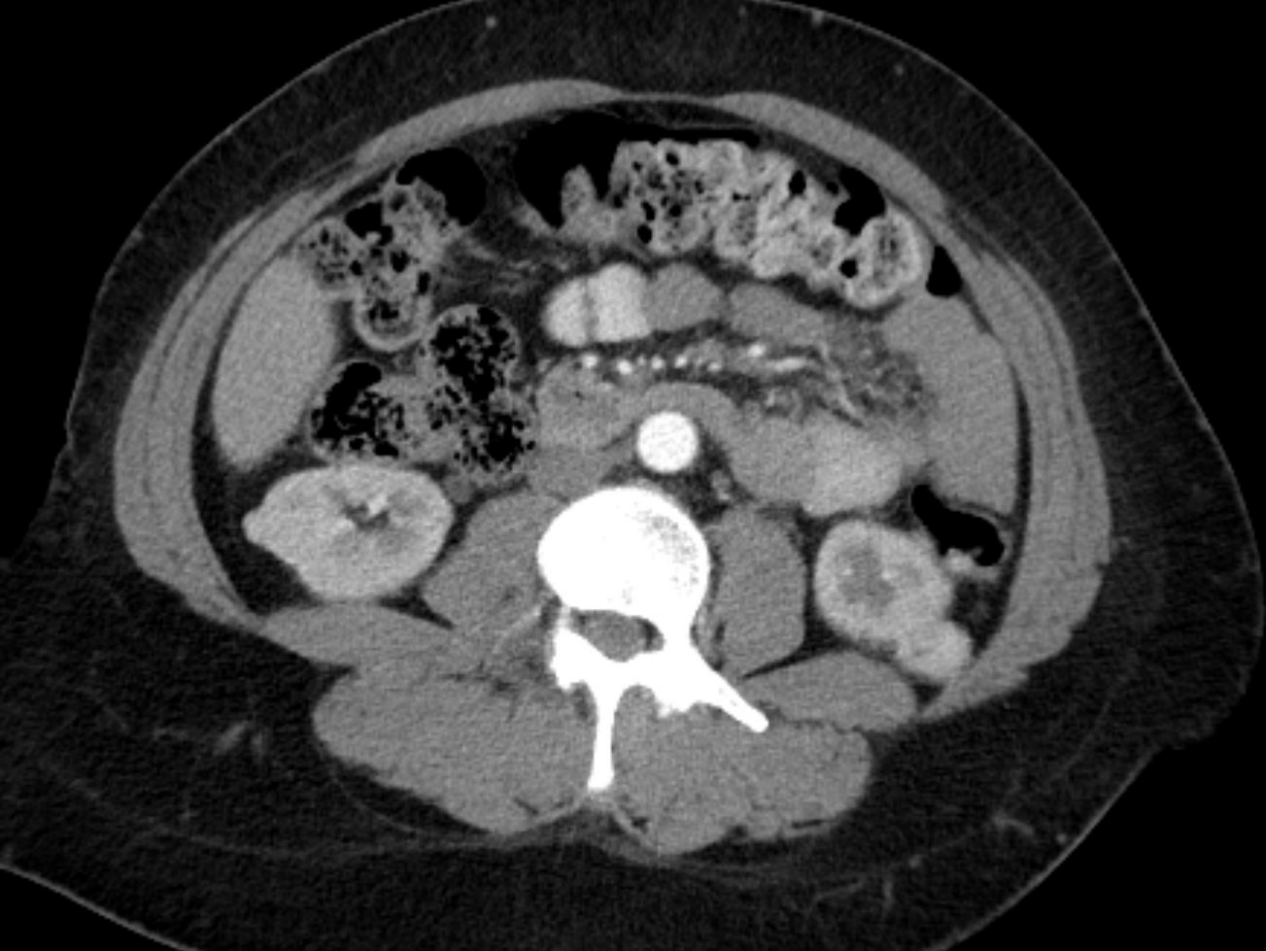
Second patient: bilateral renal tumor.

The second patient was 76 years old with a history of left open PN for ccRCC and a negative margin done 5 years ago. CT scan performed as part of postoperative surveillance diagnosed a suspected suspicious renal tumor with the left side being at the same area as that of the previous PN. Bilateral PN could be successfully done in a single stage. Pathology confirmed bilateral ccRCC, with negative margins. eGFR is stable at 12 months post-surgery. Figure 2 shows the bilateral renal tumors.

**Figure 3:**
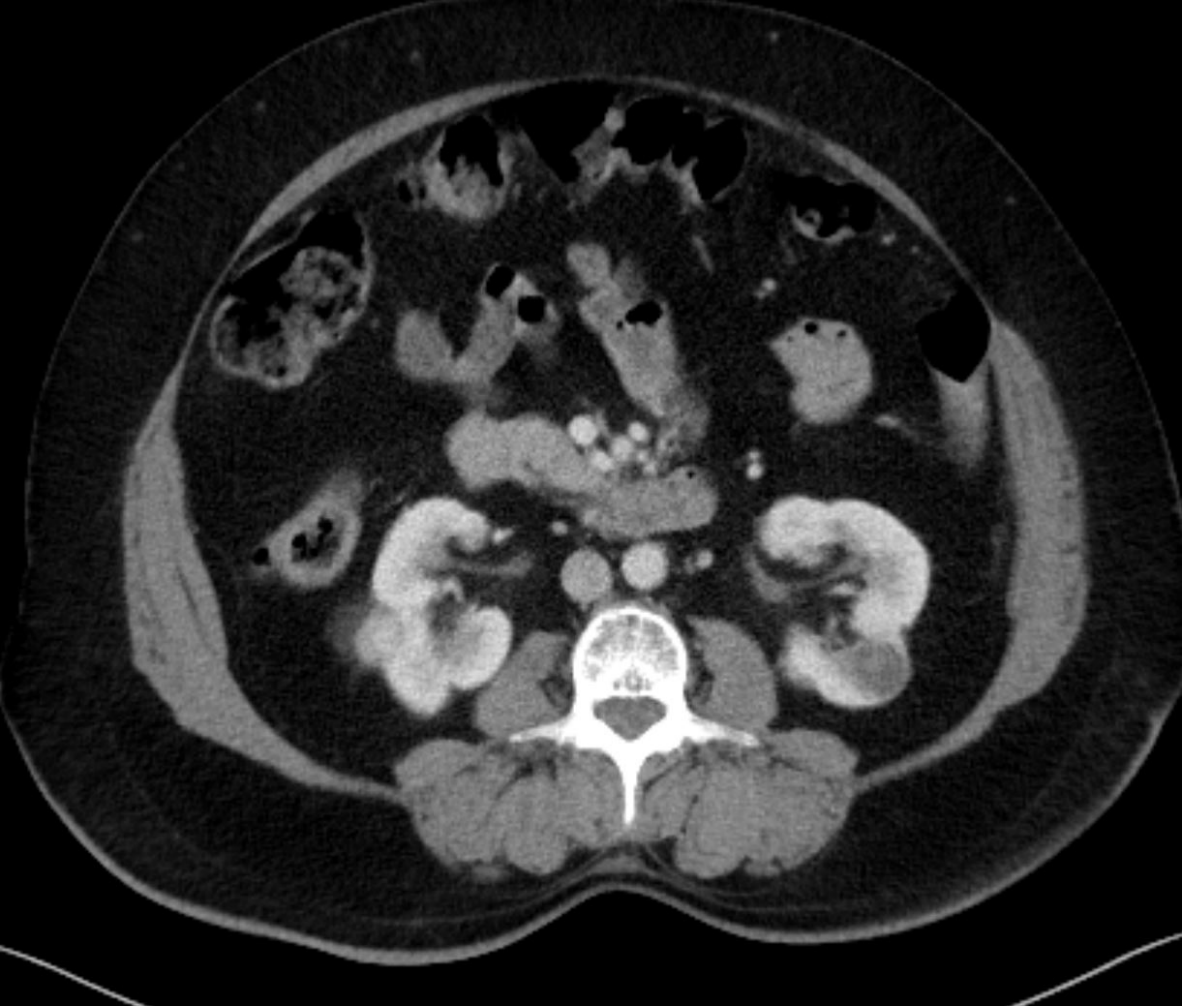
Third patient: bilateral renal tumor.

The third patient was 63 years old. He initially had a right enhanced renal mass (1.5 cm), accidentally diagnosed following an ultrasound for a vague abdominal pain. He elected for surveillance, and CT done after 1 year showed a stable right renal tumor and a new left renal tumor. Single-stage bilateral PN was done. Pathology showed bilateral ccRCC with negative margins. eGFR was stable at 12 months post-surgery. Figure 3 shows the bilateral renal tumors.

All PNs for these patients were stage T1a and were managed through single-stage bilateral transverse lateral lumbotomy. Our surgical approach has been presented in former publications (3, 4).

Warm ischemia was used for all cases undergoing PN and median warm ischemic time (WIT) was 10 min (4–16 min). The median surgical time was 120 min (90, 180, and 120 min, respectively). No blood transfusion was required during surgery or the postoperative period. The median hospital stay was 3 days (5, 3, and 2 days, respectively). We did not have any grade 3 or higher Clavien grade complications in the three patients. Table 1 summarizes patients’ clinical and pathological outcomes.

**Table 1:** Summary of patients’ clinical and pathological outcomes.

		First patient	Second patient	Third patient
Sex		Male	Male	Male
Age		78	76	63
Previous kidney surgery		No	Yes	No
Surgical approach		Bilateral transverse lateral lumbotomy	Bilateral transverse lateral lumbotomy	Bilateral transverse lateral lumbotomy
Surgery		Right PN Left RN	Bilateral PN	Bilateral PN
Total surgical time (minutes)		90	180	120
Total WIT (minutes)		4	16	10
Pathology		ccRCC and pRCC	ccRCC	ccRCC
Margins		Negative	Negative	Negative
Tumor size (cm)	Right	1.5	2	1.5
	Left	Multiple (2, 3, and 3)	2.5	2
Stage	Right	T1a	T1a	T1a
	Left	T2	T1a	T1a
Fuhrman grade	Right	3	3	3
	Left	N/A	4	2
Hospital stay		5	3	2
Preoperative eGFR		55	58	70
Postoperative eGFR		50	52	63
Clavien 3 or higher complication		None	None	None
Cancer recurrence in 1 year		None	None	None

## Discussion

Bilateral renal malignancy is infrequently encountered and the choice of single stage versus staged bilateral renal surgery is debatable and usually left to the preference of the operating surgeon. Single-stage bilateral renal surgery carries the benefit of a single anesthetic exposure in these patients with possible associated morbidities, but it is usually feared because of the anticipated higher risk of complications. Mason et al. (5) conducted a retrospective study on 76 patients who underwent single-stage bilateral PN over a period of 39 years and confirmed the safety of synchronous bilateral renal surgery, albeit with a complication rate of 20%. In this study, open surgery was performed, but the exact approach was not provided. On the other hand, Wang et al. (6) studied four patients managed by single-stage bilateral retroperitoneoscopic surgeries for renal tumors over a period of 6 years and found that four patients developed acute renal insufficiency and recommended staged surgeries rather than single stage for patients with bilateral renal tumors.

All our patients were managed through single-stage bilateral open retroperitoneal surgery through the transverse lateral lumbotomy incision. Our approach allowed easy direct access to the kidney and the renal pedicle, without the risk of pleural injury and by avoiding unnecessary exposure of the intraperitoneal organs. We usually start with the side having the larger or more difficult tumor, dissect the whole kidney and the pedicle, mark the area of resection around the tumor with cautery, and then apply warm ischemia. After removing the tumor and repairing the collecting system, we take few deep transverse mattress sutures to stop bleeding, then unclamp the pedicle and continue with renorrhaphy. We keep blood loss and WIT to the minimal, so as to avoid the risk of acute kidney injury. As our approach is subcostal, pain is well managed, better than the conventional supracostal approach, and the power of breathing is not affected in bilateral surgery as it avoids the extensive diaphragmatic dissection associated with the conventional open retroperitoneal approach. We could not identify studies that looked specifically at patients with Von Hippel–Lindau syndrome, but as renal tumors associated with this syndrome tend to grow faster, single-stage bilateral surgery should be considered whenever feasible.

Renal biopsy is generally not recommended for enhanced renal mass before surgery, unless some other pathology is suspected or a minimally invasive intervention like radiofrequency ablation is planned. As our three patients elected for surgery and CT clearly showed enhanced heterogenous masses, surgery was directly planned.

Klatte et al. (7) conducted an international multicentric study from 12 urological centers studying 118 patients who presented with synchronous bilateral RCC. Half of the patients were managed through a single-stage bilateral surgery. The authors confirmed equal oncological and functional outcomes. They added that synchronous renal RCC tends to have a higher frequency of papillary histology than unilateral cases. A large review of the SEER database for patients with bilateral solid renal tumors found a malignant concordance rate of 99% and a histological concordance rate of 93% (8). In our series, one out of three patients had histological dis-concordance with clear-cell histology identified in one kidney and papillary histology in the other kidney. Arnoux et al. (9) found that ccRCC is associated with pRCC in 3% of cases.

Reddy et al. (10) reported grade 3 or higher complications in nearly 15% of their cases managed by PN, mostly manifested in the form of urine leak or bleeding requiring exploration or embolization. We did not encounter these complications in any of the three patients.

The main limitation of our report is the less number of patients; however, this together with the available few other reports may confirm the safety of single-stage bilateral renal cancer surgery. Although Canadian urological association guidelines recommend first postoperative CT to be done at 24 months (11), we elected to do it at 12 months considering the possible higher risk of recurrence attributed to synchronous bilateral cancer. CT failed to identify any recurrent disease in all patients.

## Conclusion

Single-stage bilateral renal surgery is a safe procedure in experienced hands. Bilateral transverse lateral lumbotomy allows for a fast and safe surgery with minimal complications. There is a possible histological dis-concordance in bilateral synchronous RCC.
